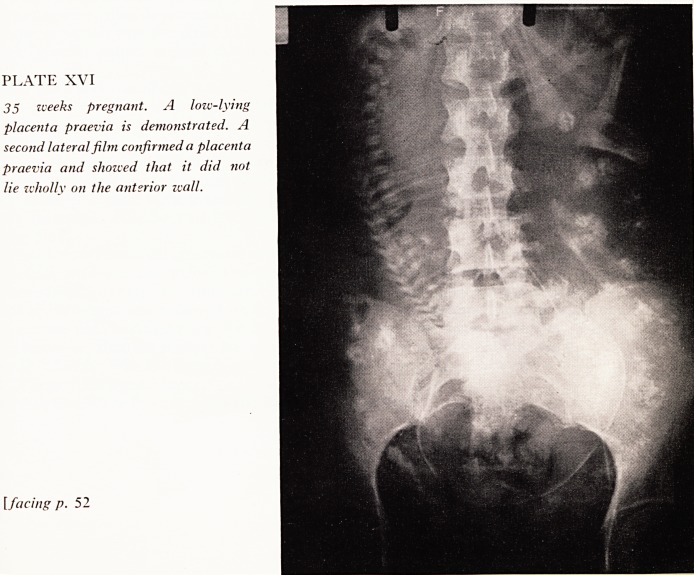# Placental Localization by Arteriography

**Published:** 1965-07

**Authors:** M. B. Wingate, N. D. O'Connell

**Affiliations:** Southmead Hospital, Bristol; Southmead Hospital, Bristol


### PLACENTAL LOCALIZATION BY ARTERIOGRAPHY

BY

M. B. WINGATE AND N. D. O'CONNELL

*Southmead Hospital, Bristol*

At the present time the only certain method of diagnosing placenta praevia in a pregnant woman who has had an ante-partum haemorrhage is by examination under anaesthesia. The examination is usually deferred until the 38th week of pregnancy, when the foetus is considered to be sufficiently mature should premature delivery have to be effected. The procedure is traumatic and may induce labour with or without further haemorrhage.

The patient may have presented herself with ante-partum haemorrhage some 4 or more weeks before this time, and many such patients are at present occupying ante-natal beds in major obstetric units. The prolonged period of hospital stay is often a cause of considerable domestic difficulty and at the end of that time no placenta praevia may be found and the patient is discharged to await the onset of normal labour.

For these reasons obstetricians have looked for a method of placental localization which could be used at an earlier stage of pregnancy without danger to mother or baby. The problem has been the subject of a recent editorial review in the *British Medical Journal* (1964). Many radiological methods to aid placental localization have been described, and include soft tissue studies, use of the hypaque pillow and differential filters, contrast filling of the bladder and rectum, placental calcification, amniography isotope studies with iodine-labelled serum albumen, and arteriography. Ultrasonic (*B.M.J.* 1964) and thermographic (Young 1964) techniques are at present the subject of intensive research. Since August 1963 we have used arteriography at Southmead Hospital on selected patients and our experience in the ten cases so examined is now reviewed.

Arteriography as a method of demonstrating the placental site has been advocated for several years. Sutton (1962) described a method employing forced percutaneous needle injection of the femoral artery, and aided retrograde filling of the iliac arteries by pressure cuffs around both thighs and the performance of the Valsalva manoeuvre. Hartnell (1948) published an account of sixty-eight cases using the translumbar approach under general anaesthesia, but the procedure could not be considered to have become a comparatively simple one until Seldinger (1953) described his technique. Since that time several series have been published of which the most notable is by Fernstrom (1955). All these series claim uniformly successful results and few complications.

### MATERIAL AND METHOD

Ten patients with ante-partum haemorrhage, suspected on clinical grounds to be due to placenta praevia, have been investigated. The examinations were carried out between the 32nd and 36th weeks of pregnancy.

The Seldinger technique is a relatively simple one. The equipment consists of a flanged cannula with a smooth straight margin and a removable stilette which projects a little way beyond the margin, a thin metal guide wire with a flexible tip, and a polythene or opaque catheter which has a tap at its distal end.

The area of skin over the femoral artery and lying immediately below the inguinal ligament is infiltrated with a local anaesthetic, and the cannula and stilette are introduced into the artery. The stilette is removed, and pulsating bright red blood indicates that the cannula lies within the vessel. The guide wire is now introduced and passed along the cannula some distance into the lumen of the vessel. The flexible tip is of great help in negotiating the angle between the cannula and the artery wall.

The cannula is now withdrawn and the catheter which is closely applied to the guide wire is passed along the wire into the artery, the wire itself is now withdrawn leaving the catheter in the artery. The tip of the catheter must be positioned just proximal to the aortic bifurcation and an image intensifier and television screen are used to ensure correct positioning of the catheter and to reduce radiation to a minimum. Should a polythene catheter be used contrast medium is needed to opacify it.

Twenty millilitres of contrast medium are now injected, and one antero-posterior (A.P.) film of the abdomen is taken about four seconds after the end of the injection. The placenta can be readily visualized, the sinusoids being outlined by contrast medium as multiple irregular woolly areas (Plate XV). The timing of the film is most important and two early cases in our series required two films as the sinusoids were not well shown on the first film. With increased experience only one film becomes necessary, but it must be noted that when a low-lying placenta is found in the A.P. film, confirmation that it is praevia must be obtained by a lateral film.

### RESULTS

We failed to catheterize one patient but the placenta was clearly demonstrated in the other nine patients. One placenta praevia was found (Plate XVI) and confirmation was obtained when this patient was delivered by lower segment caesarean section. Another patient was discharged but is not yet at term. Of the remaining seven patients, five were discharged following investigation, and re-admitted at or about term when in labour. All these patients had normal deliveries. Two had premature deliveries at 36 weeks of pregnancy. The interval between arteriography and delivery in one of these patients was a week, but in the second patient only 3 days. This latter patient had recurrent haemorrhages during the pregnancy, at 25 weeks, then at 33 weeks, again at 34 weeks, but no bleeding for the 2 days immediately before the arteriogram. She had a further bleeding episode on the day following the examination, and on the day of delivery.

### COMPLICATIONS

One patient had thrombosis of the femoral vein on the side of the catheterization 7 days after the examination and 4 days after delivery. There was some venous oozing at the time of the injection, indicating accidental venous puncture, but examination on the following morning revealed no bruising or haematoma formation. The patient was delivered in stirrups and it may be that the summation of trauma caused the thrombosis, which however cleared quickly and completely.

Reference has already been made on the failure to catheterize one patient. This was due to arterial spasm and while it is now our practice to attempt the opposite side should the first attempt be unsuccessful, this patient was one of the earliest cases to be investigated and we were unsure of the technique. The second attempt was postponed and did not in fact take place because of domestic complications. She was later delivered by caesarean section for a type II placenta praevia.

The first patient was catheterized under general anaesthesia, and vomited on several occasions on the day following the examination, and drained liquor for 2 days. She was discharged to return at term, when she had a normal spontaneous vaginal delivery. Since that time all examinations have been carried out under local anaesthesia. A small haematoma formed at the injection site in one case, but gave rise to no ill effects and was quickly absorbed.

### DISCUSSION

The question of the safety of arterial puncture has recently been discussed by Hoskin (1964). Excluding direct trans-lumbar aortic puncture, where the incidence of complications is high save in the most experienced hands, the incidence of complication in other forms of arterial puncture is low. Complications reported have included haemorrhage from the puncture wound, femoral arterial and venous thrombosis, haematoma formation and occasionally abscess formation. The actual incidence is however low and many large series have been reported without any complications at all.

### RADIATION

The exact radiation dosage involved in the investigation is not known as this requires accurate measurement during the actual examination. However, an approximate estimate of the dose can be given. Radiation is produced initially during the screening of the catheter into position and later, at the exposure of the A.P. film of the abdomen. The screening time in the former is about 8–10 seconds, which should produce a skin dosage of approximately 300 mr (Gregg, Allcock and Berridge, 1957). The average dose to the maternal gonads in a pregnant patient is 260 mr (Stanford and Vance, 1955) during a single A.P. abdominal film. The dose is kept to a minimum by using one A.P. film only and by the very short screening time, made possible by using the Image Intensifier and television units.

### SUMMARY

The conservative management of a pregnant patient suspected of having a placenta praevia often involves a prolonged stay in hospital. This may give rise to domestic and economic difficulties. A method of demonstrating the placental site by arteriography has been presented, together with a small series of cases in which the procedure has been used. With increasing experience it is felt that femoral arteriography is a comparatively simple and safe method of demonstrating the presence or absence of a placenta praevia from the 32nd week of pregnancy.

### REFERENCES

Fernstrom, I. (1955). *Acta Radiol. Suppl.*, 122.
Gregg, D. McC., Allcock, J. M., Berridge, F. R. (1957). *Brit. J. Radiol.*, **30**, 423.
Harnett, L. J. (1948). *Amen. J. Obstet. and Gynec.*, **55**, 940.
Placental Localization (1964). *Brit. Med. J.*, **2**, 261.
Seldinger, S. I. (1953). *Acta Radiol. Stockh.*, **39**, 368.
Stanford, R. W., Vance, J. (1955). *Brit. J. Radiol.*, **38**, 266.
Sutton, D. (1952). *Brit. J. Radiol.*, **25**, 320.
Ultrasonic Diagnosis (1964). *Brit. Med. J.*, **2**, 402.
Young, R. T. (1964). *Brit. Med. J.*, **2**, 978.

PLATE XV

*34 weeks pregnant. The placenta is shown outlined with contrast in the right upper lateral wall of the uterus.*

PLATE XVI

*35 weeks pregnant. A low-lying placenta praevia is demonstrated. A second lateral film confirmed a placenta praevia and showed that it did not lie wholly on the anterior wall.*

## Figures and Tables

**PLATE XV f1:**
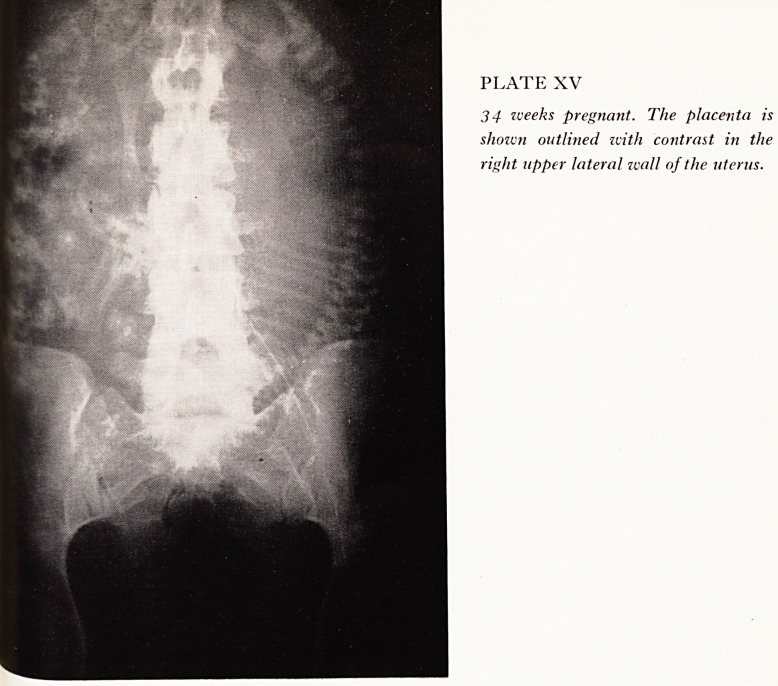


**PLATE XVI f2:**